# Second-Line Gemcitabine Plus Nab-Paclitaxel for Patients with Unresectable Advanced Pancreatic Cancer after First-Line FOLFIRINOX Failure

**DOI:** 10.3390/jcm8060761

**Published:** 2019-05-29

**Authors:** Naoki Mita, Takuji Iwashita, Shinya Uemura, Kensaku Yoshida, Yuhei Iwasa, Nobuhiro Ando, Keisuke Iwata, Mitsuru Okuno, Tsuyoshi Mukai, Masahito Shimizu

**Affiliations:** 1 First Department of Internal Medicine, Gifu University Hospital, 1-1 Yanagido, Gifu 501-1194, Japan; mitanao8@yahoo.co.jp (N.M.); ueshin550621@gmail.com (S.U.); kensakuyoshidaky@gmail.com (K.Y.); festinalenteyu@gmail.com (Y.I.); shimim-gif@umin.ac.jp (M.S.); 2 Department of Gastroenterology, Gifu Prefectural General Medical Center, Gifu 500-8717, Japan; nobuhiro5091ando@yahoo.co.jp (N.A.); keisukeiwata@nifty.com (K.I.); 3 Department of Gastroenterology, Gifu Municipal Hospital, Gifu 500-8513, Japan; mkobdkl@yahoo.co.jp (M.O.); tsuyomukai@yahoo.co.jp (T.M.)

**Keywords:** pancreatic cancer, second-line chemotherapy, gemcitabine plus nab-paclitaxel, FORFILINOX

## Abstract

FOLFIRINOX (FX) and gemcitabine (GEM) plus nab-paclitaxel (GnP) have been reported as effective regimens for unresectable advanced pancreatic cancer (APC). FX may be more effective but is also associated with more adverse events (AEs). Therefore, first-line treatment with FX followed by second-line GnP may be appropriate. Aims: To assess the safety and efficacy of second-line GnP for patients with APC after first-line FX failure. Methods: This study was a multicenter prospective phase II study evaluating second-line GnP in patients with APC after failed first-line FX. The primary endpoint was response rate (RR), and the secondary endpoints were overall survival (OS), progression free survival (PFS), and the frequency and degree of adverse events (AEs). Results: Thirty patients (14 male; median age, 64 years) were enrolled. The RR was 13.3%, with a median follow-up time of 9.3 months. The median OS and PFS were 7.6 and 3.8 months, respectively. From the beginning of first-line treatment, the median OS and PFS were 14.2 and 9.3 months, respectively. Grade 3 or 4 AEs were seen in 70% of patients. Conclusion: Second-line GnP after FX failure for patients with APC could be more effective than GEM alone. Further comparison studies are warranted.

## 1. Introduction

Pancreatic cancer is the seventh leading cause of cancer-related death, with more than 330,000 deaths worldwide annually [1]. Patients with pancreatic cancer have a poor prognosis, and the overall five-year survival rate is only 8%, since pancreatic cancer is often diagnosed in an advanced stage with locally-advanced and/or metastatic lesions [2]. Therefore, the development of an effective and tolerable chemotherapy regimen is crucial for improving the outcomes of patients with advanced pancreatic cancer (APC). Gemcitabine (GEM) has been the standard treatment for pancreatic cancer since the late 1990s. Recently, two regimens, FOLFIRINOX (FX; a combination of 5-fluorouracil, oxaliplatin, irinotecan, and leucovorin) and GEM plus nab-paclitaxel (GnP), have shown better outcomes than GEM alone in terms of overall survival (OS), progression survival (PFS), and response rate (RR) [3,4].

The development of FX and GnP has increased treatment options for patients with APC and raised concerns about the selection and sequence of treatments. Considering that FX, which is a four-agent combination therapy, causes more adverse events (AEs) than GnP, especially in Japanese cohorts, FX indications tend to be more limited and include better performance status (PS) and younger age than those of GnP. Furthermore, according to the phase III studies of FX and GnP [3,4], the OS, PFS, and RR are slightly better with FX. Considering these points, one possible strategy for APC treatment could involve first-line FX followed by second-line GnP. To evaluate this strategy, we conducted a multi-center prospective phase-II study of second-line GnP for patients with APC after FX treatment failure. 

## 2. Patients and Methods

### 2.1. Study Design

This study was designed as an open-label, multicenter prospective phase II study of second-line GnP after failure of FX for unresectable APC. The primary endpoint was the RR. The secondary endpoints were OS, PFS, and the frequency and degree of AEs. All patients provided written informed consent. The study was carried out in accordance with the Declaration of Helsinki. The study protocol was registered with University hospital Medical Information Network Clinical Trials Registry (ID: UMIN000016624).

### 2.2. Patient Eligibility Criteria

Patients were eligible if they met the following criteria: pathologically proven adenocarcinoma or adenosquamous carcinoma of the pancreas; unresectable APC, including locally-advanced and metastatic cancer; failure of FX therapy, including modified regimens (oxaliplatin intravenously at 85 mg/m^2^ for 2 h, leucovorin intravenously at 400 mg/m^2^ for 2 h, irinotecan intravenously at 150 mg/m^2^ for 90 min, and fluorouracil (5-FU) intravenously at 2400 mg/m^2^ over 46 h) [5] as the first-line chemotherapy; age of 20–75 years; Eastern Cooperative Oncology Group PS score of 0 or 1; peripheral neuropathy of ≤grade 1 (loss of deep tendon reflexes or paresthesia) based on Common Terminology Criteria for Adverse Events (CTCAE) v4.0; possible oral intake; no electrocardiographic abnormalities within 4 weeks of protocol entry; absolute neutrophil count >1500/mm^3^, hemoglobin level >9.0 g/dL, platelet count >100,000/mm^3^, total bilirubin level <2.0 mg/dL, aspartate transaminase and alanine transaminase levels <150 U/L, and creatinine level <1.5 mg/dL. Patients were excluded from the study if they met any of the following conditions: pulmonary fibrosis or interstitial pneumonia; watery stools; active infection; serious concomitant diseases; apparent pleural effusion or ascites; metastasis to the central nervous system; synchronous double cancer or metachronous double cancer with a disease-free period of less than 3 years; patients who were pregnant, lactating, or planning a pregnancy, and men who desired pregnancy of their partners; those with serious mental disorders; and anyone considered ineligible by the investigators.

### 2.3. Treatment

At first, patients received dexamethasone 6.6 mg intravenously for 15 min as an antiemetic; after which, nab-paclitaxel was injected intravenously at 125 mg/m^2^ for 30 min, followed by intravenous gemcitabine at 1000 mg/m^2^ for 30 min. Initial doses of nab-paclitaxel and gemcitabine could be reduced to those of level 1 at the attending doctor’s discretion, based on the condition of the patient (Table 1). These drugs were administered on days 1, 8, and 15. The treatment was continued in repeating 28-day cycles as long as the regimen was tolerated and/or until disease progression, discontinuation decided by the investigators, or patient refusal. For careful safety evaluations, all patients were admitted to the hospital at the initiation the first GnP cycle. Granulocyte-colony stimulating factor (G-CSF) was not administered as a prophylaxis against neutropenia or febrile neutropenia (FN).

Dose reductions or treatment delays were performed as described for the first-line treatment [4]. The treatment was delayed in cases of one or more of the following at day 1 of any cycles: white blood cell count (WBC) count >12,000/mm^3^, absolute neutrophil count <1500/mm^3^, hemoglobin level <9.0 g/dL, platelet count <100,000/mm^3^, total bilirubin level >2.0 mg/dL, aspartate transaminase and alanine transaminase levels >150 U/L, creatinine level >1.5 mg/dL, peripheral neuropathy, FN. The treatment was skipped if one or more of the following occurred at days 8 and 15: absolute neutrophil count <500/mm^3^, platelet count <50,000/mm^3^, grade ≥3 peripheral neuropathy, FN, grade ≥3 oral mucositis, and grade ≥3 diarrhea. The doses of nab-paclitaxel and GEM were reduced if absolute neutrophil count reached 500–1000/mm^3^ and/or platelet count reached 50,000–75,000/mm^3^ at day 8 or 15. Dose reductions due to non-hematological toxicities were performed as follows: if grade ≥2 exanthema or grade ≥3 oral mucositis or grade ≥3 diarrhea developed, both nab-paclitaxel and GEM were reduced, and if grade ≥3 peripheral neuropathy developed, only nab-paclitaxel was reduced.

### 2.4. Assessment 

Throughout the entire treatment course, patients were assessed for their general condition and any possible adverse events, by physical and blood examinations that included complete blood counts and blood chemical tests. The examinations were generally performed once a week by the attending physicians. The treatment response was assessed by the radiologists at each center, by comparing the computed tomography scans that were taken at baseline to the scans that were taken at least every 12 weeks after treatment initiation. Adverse events were scored using the National Cancer Institute Common Terminology Criteria for Adverse Events version 4.0. The radiologic tumor response was evaluated using the Response Evaluation Criteria in Solid Tumors version 1.0.

### 2.5. Statistical Analysis

The RRs were defined as the best observed RRs. OS was calculated from the date of second-line GnP or first-line modified FX (mFX) initiation to the date of death. PFS was calculated from the date of second-line GnP initiation to the date of disease progression. The relative dose intensity was calculated as the ratio of the amount of drug that was actually administered to the amount of standard regimen during the whole treatment period, from the date of GnP initiation to completion. The OS, PFS, and RR outcomes were calculated, with the corresponding 95% confidence intervals (CIs). OS and PFS were estimated using the Kaplan–Meier method. All statistical analyses were performed using JMP 10.0 (SAS Institute, Inc, Cary, NC, USA). Since the RRs of second-line GEM and GnP for APC were barely unknown, the power calculation was performed based on the results of the phase III study of GnP. Considering some deterioration of RRs from those in the phase III study, the RR of second-line GEM and GnP were assumed as 5% and 20%, respectively. The RR threshold and the expected RR were then set at 5% and 20%, respectively. Subsequently, the required sample size was calculated as 27 patients, by a one-arm binomial sample size calculation, with a power of at least 90% and a one-sided significance level of 5%. Accordingly, the target sample size was set as 30 patients, to account for an omission rate of 10%.

## 3. Results

### 3.1. Patient Characteristics

**Figure 1:**
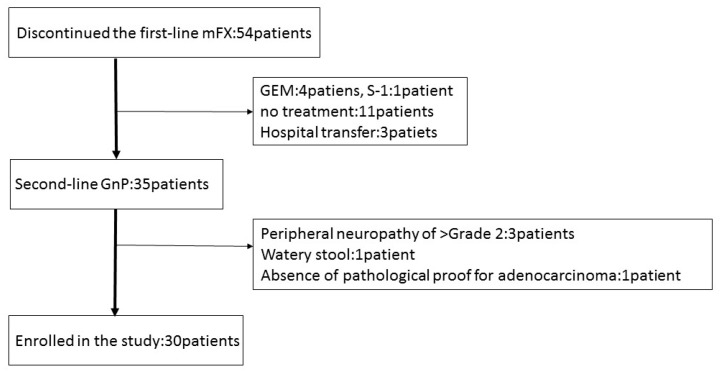
Patient flow chart. mFX, modified FOLFIRINOX; GEM, gemcitabine; S-1, the combination drug of tegafur, gimeracil and oteracil; GnP, gemcitabine plus nab-paclitaxel.

Between January 2015 and May 2017, 54 patients discontinued first-line mFX due to primary disease progression (46), adverse event (5); anorexia, peripheral neuropathy, drug allergy, neutropenia, pulmonary fibrosis), and deterioration of the PS (3). Thirty-five patients received GnP as second-line treatment, five of whom were excluded due to peripheral neuropathy ≥grade 2, watery stool, and absence of pathological proof for adenocarcinoma or adenosquamous carcinoma. Thirty patients were recruited and enrolled into this study at Gifu University Hospital, Gifu Municipal Hospital, and Gifu Prefectural General Medical Center (Figure 1). The basic patient characteristics are shown in Table 2. The first-line mFX therapy was interrupted due to primary disease progression in 29 patients and due to adverse event (drug allergy) in one patient. There were a total of eight FX cycles (range, 2–35), with best responses of partial response (PR) in seven patients (23.3%), stable disease (SD) in 12 patients (40%), and progressive disease (PD) in 11 patients (36.7%).

### 3.2. Actual GnP Treatment

**Figure 2:**
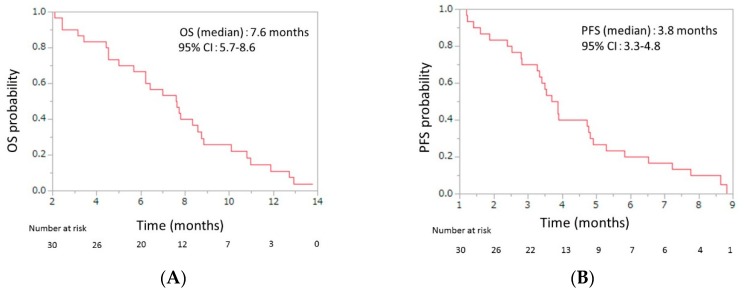
(**A**) Overall survival (OS) and (**B**) progression-free survival (PFS) of patients with unresectable advanced pancreatic cancer who were treated with second-line gemcitabine plus nab-paclitaxel after FOLFIRINOX failure. The median OS was 7.6 months (95% confidence interval (CI), 5.7–8.6), and the median PFS was 3.8 months (95% CI, 3.3–4.8).

**Figure 3:**
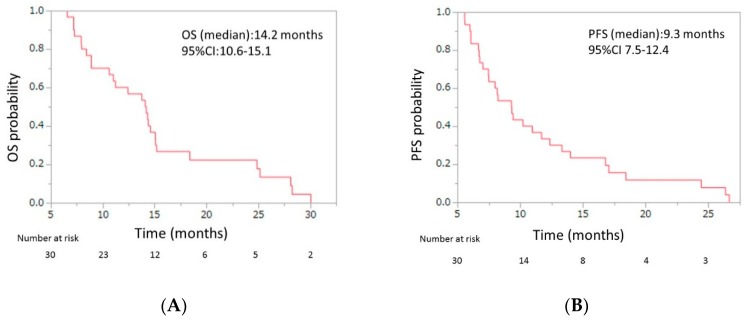
(**A**) Overall survival (OS) and (**B**) progression-free survival (PFS) of patients with unresectable APC who were treated with second-line gemcitabine plus nab-paclitaxel after FOLFIRINOX (FX) failure, from beginning of the first-line FX. The median OS was 14.2 months (95% confidence interval (CI), 10.6–15.1), and the median PFS was 9.3 months (95% CI, 7.5–12.4). APC, advanced pancreatic cancer.

Initial dose reduction was performed in seven patients. The median number of cycles was four (range, 2–13), and the median relative dose intensities were as the follows: nab-paclitaxel, 67.0% (range 42.3–100); GEM 72.3% (range 42.9–100) (Table 3). The response evaluations showed a partial response in four patients (13.3%), stable disease in 10 patients (33.3%), and progressive disease in 16 patients (53.3%). The response rate was 13.3% (95% CI, 5.3–29.7), and the disease control rate was 46.7% (95% CI, 30.2–63.9) (Table 4). The median OS was 7.6 months (95% CI, 5.7–8.6), and the median PFS was 3.8 months (95% CI, 3.3–4.8) (Figure 2). From the beginning of first-line mFX, the median OS was 14.2 months (95% CI, 10.6–15.1) (Figure 3).

### 3.3. Safety Profile and Third-Line Treatment

Twenty-one (70%) of the 30 patients experienced grade ≥3 adverse events. The hematologic adverse events included neutropenia in 15 patients (50%), febrile neutropenia in two (6.7%), thrombocytopenia in six (20%), and anemia in eight (26.7%). The grade ≥3 non-hematologic adverse events included peripheral sensory neuropathy in four patients (13.3%), anorexia in four (13.3%), and diarrhea in one (3.3%) (Table 5). At the time of the analysis, second-line treatment was still ongoing in one patient and was discontinued in 29 patients due to progressive disease in 23 patients, adverse events (pulmonary fibrosis) in three patients, and deterioration of PS in three patients. Chemotherapy-related death did not occur. Third-line treatment using the combination drug of tegafur, gimeracil and oteracil (S-1) was performed in 15 patients, although the remaining 14 did not receive further chemotherapy due to deterioration of their conditions (Table 6).

## 4. Discussion

This phase II prospective pilot study evaluated second-line GnP for patients with APC after first-line FX failure and found an RR of 13.3% (95% CI, 5.3–29.7), disease control rate (DCR) of 46.7% (95% CI, 30.2–63.9), median OS of 7.6 months (95% CI, 5.7–8.6), and PFS of 3.8 months (95% CI, 3.3–4.8), over a median follow-up time of 9.3 months. From the initiation of the first-line treatment, the median OS was 14.2 months (95% CI, 10.6–15.1). Grade 3 or 4 adverse events included neutropenia (50%), FN (6.7%), thrombocytopenia (20%), anemia (26.7%), peripheral sensory neuropathy (13.3%), anorexia (13.3%), and diarrhea (3.3%).

A comprehensive analysis of second-line treatments in patients with APC found that second-line chemotherapies for APC were preferred over the best supportive care, even before the development of FX and GnP regimens [6]. With respect to second-line therapy after failed FX, several studies have evaluated the efficacy of second-line GEM alone, which showed RR, DCR, median OS, and median PFS values of 10–11%, 26–40%, 3.6–5.7, and 1.5–2.5 months, respectively (Table 7) [7,8,9,10]. The treatment outcomes of second-line GnP after failed FX in our study tended to be worse in comparison to those reported in the original phase III study of first-line GnP: RR of 23 %, DCR of 48%, and median OS of 8.5 months [4]. As for second-line GnP after failed FX, one report included 57 patients with metastatic APC, and showed an RR of 17%, DCR 58%, median OS of 8.8 months, and median PFS of 5.1 months [11]. Our results from second-line GnP after failed FX tended to be better than those of second-line GEM alone and were similar to those reported for second-line GnP. Considering these results, second-line GnP after failed FX could be considered a more effective regimen than GEM alone, although these tendencies should be cautiously interpreted because of possible differences in patient characteristics.

Regarding the toxicity profiles that were observed in this study, there were no obvious differences in hematological adverse event rates between our phase II second-line GnP and Japanese phase II study or phase III study of first-line GnP [4,12]. The rates of grade 3 or 4 neutropenia and FN were 50% and 6.7%, respectively, lower or similar to those (70.6% and 5.9%, respectively) reported in a phase II study of first-line GnP, and higher than those (38% and 3%) of the phase III study of GnP. No patients discontinued treatment due to hematological adverse events in our study. With respect to non-hematological adverse events, the rate of grade 3 or 4 peripheral sensory neuropathy (13.3%) in our study was similar to that of Japanese phase II and phase III studies (11.8% and 17%). However, three patients (10%) discontinued second-line GnP due to grade 2 interstitial pneumonia in our study. Although the exact reasons for the higher interstitial pneumonia rate were not clear, it might be important to carefully observe respiratory conditions in second-line GnP therapy after failed FX.

Given the efficacy and safety profiles of second-line GnP discussed above, first-line FX followed by second-line GnP could be an effective treatment regimen for patients with APC. Notably, the median OS from the initiation of first-line FX in our study was tended to be longer than those in the original phase III studies of FX and GnP. Another possible strategy with the same concept might involve first-line GnP followed by second-line FX; however, considering that FX combines four agents and causes more AEs, especially hematological AEs and FN, GnP followed by FX might result in unfavorable outcomes. Furthermore, a recent population-based study [13] that included 693 patients with APC, which was conducted after FX and GnP became available, reported that first-line FX was the strongest factor associated with administration of second-line chemotherapy.

Recently, novel treatment agents, such as immune check point inhibitor (ICI) [14] or poly ADP-ribose polymerase (PARP) inhibitor [15], have been reported as effective treatment regimens for APC. However, indications of these agents are limited: pembrolizumab which is one of ICIs has indication only for micro satellite instability-high being around 1–2% of APC patients [16] and PARP inhibitor has an indication for BRCA 1/2 mutation being up to 10% of APC [17]. Even with limited indications, those agents potentially can improve the treatment outcomes of patients with APC. Since limited data is available for ICIs and PARP inhibitors for APC, further evaluations of those new agents await, including efficacy and safety as second-line treatments of APC.

This study has several limitations. First, we included a small number of patients and only three centers, which might cause bias in patient selection and decrease external validity. Second, because our study lacked a comparison arm, further comparison studies between GnP and other second-line regimens might be required to confirm our study findings and decide the ideal treatment strategy for APC. Third, our study included patients with metastatic and locally advanced pancreatic cancers, which might affect study outcomes, especially in terms of OS. Fourth, initial dose modifications were allowed in this study, which might affect the study outcomes, especially with respect to safety. The strength of this study was the prospective multicenter study design and the initial evaluation of second-line GnP after failed FX in patients with APC.

In conclusion, this open-labeled phase II study showed that second-line GnP after failed mFX therapy in Japanese patients with APC including locally advanced and metastatic cancer was feasible, with decent efficacy and safety. Further large-scale comparison studies are required to confirm the efficacy and safety of second-line GnP and the performance of second-line GnP after first-line FX failure in patients with APC.

## Figures and Tables

**Table 1:** Dose modifications.

Dose Level	Nab-Paclitaxel (mg/m^2^)	Gemcitabine (mg/m^2^)
Full Dose	125	1000
Level 1	100	800
Level 2	75	600

**Table 2:** Baseline characteristics of patients with unresectable advanced pancreatic cancer (APC) treated with second-line gemcitabine plus nab-paclitaxel (GnP) after failed FOLFIRINOX (FX).

Characteristics		*n* = 30	
		*n*	%
**Age**	Median (range)	64 (37–71)	
**Sex**	Male/Female	14/16	
**ECOG-PS**	0/1	24/6	80/20
**CA19-9**	Median (range)	1952.2 (14.5–81897)	
**Disease extent**	Locally advanced	6	20
	Metastasis	24	80
**Site of primary tumor**	Head/Others	14/16	46.7/53.3
**Site of metastasis**	Liver/Lung/Others	20/4/4	66.7/13.3/13.3
**Biliary drainage**	Yes/No	12/18	40/60
**Number of mFX cycles**	Median (range)	8 (2–35)	
**Response of mFX**	CR	0	0
	PR	7	23.3
	SD	12	40
	PD	11	36.7
**Reasons for mFX interruption**	PD	29	96.7
	Adverse events	1	3.3

ECOG PS, Eastern Cooperative Oncology Group Performance Status; CA19-9, charbohydrate antigen 19-9; mFX, modified FOLFIRINOX; CR, complete response; PR, partial response; SD, stable disease; PD, progressive disease.

**Table 3:** Treatment duration and drug delivery of patients with unresectable APC treated with second-line gemcitabine plus nab-paclitaxel after failed FOLFIRINOX.

		*n* (Range)	
Median cycles of treatment		4 (2–13)	
**Median relative dose intensity**		**% (range)**	
nab-PTX		67.0 (42.3–97.2)	
GEM		72.3 (42.9–100)	
**Initial dose**		*n*	**%**
nab-PTX	100%	23	76.7
	80%	7	23.3
GEM	100%	24	80
	80%	6	20

nab-PTX, nab-paclitaxel; GEM, gemcitabine.

**Table 4:** Efficacy results of patients with unresectable advanced pancreatic cancer treated with second-line gemcitabine plus nab-paclitaxel after failed FOLFIRINOX.

Efficacy	*n*	%	95% CI
**Complete response**	0	0	
**Partial response**	4	13.3	
**Stable disease**	10	33.3	
**Progressive disease**	16	53.3	
**Response rate**	4	13.3	5.3–29.7
**Disease control rate**	14	46.7	30.2–63.9

95% CI: 95% confidence interval.

**Table 5:** Adverse events of patients with patients with unresectable APC treated with second-line gemcitabine plus nab-paclitaxel after failed FOLFIRINOX.

Adverse Events	Total		≧Grade 3	
	*n*	%	*n*	%
**Hematological toxicities**				
Neutropenia	22	73.3	15	50
Febrile neutropenia	2	6.7	2	6.7
Thrombocytopenia	9	30	6	20
Anemia	16	53.3	8	26.7
**Non-hematological toxicities**				
Nausea and vomiting	8	26.7	0	0
Peripheral sensory neuropathy	13	43.3	4	13.3
Alopecia	4	13.3	0	0
Anorexia	7	23.3	4	13.3
Dysgeusia	4	13.3	0	0
Mucositis oral	3	10	0	0
Fatigue	3	10	0	0
Erythema	2	6.7	0	0
Diarrhea	2	6.7	1	3.3
Pulmonary fibrosis	3	10	0	0

APC, advanced pancreatic cancer.

**Table 6:** Continuation of second-line treatment and third-line treatment.

Second-line treatment	*n*
Ongoing	1
Discontinued	29
Reasons for discontinuation	
Progression disease	23
Adverse events (Pulmonary fibrosis)	3
Decline in the performance status	3
Chemotherapy related death	0
**Third-line treatment**	
S-1	15
No treatment	14

S-1, the combination drug of tegafur, gimeracil and oteracil.

**Table 7:** Previous studies about second-line gemcitabine (GEM) alone after failed FX.

Author Year	Number of Patients	RR (%)	DCR (%)	Median OS (months)	Median PFS (months)
da Rocha Lino, A. et al., 2015 [7]	20	NA	NA	5.7	2.0
Viaud, J. et al., 2017 [8]	80	10	40	3.7	2.1
Gilabert, M. et al., 2017 [9]	72	11	35	NA	2.5
Sarabi, M., 2017 [10]	42	NA	26	3.6	1.5

GEM, gemcitabine; FX, FOLFIRINOX; OS, overall survival; DCR, disease control rate; PFS, progression free survival; NA, not applicable.

## Abbreviations

**Abbreviations**	**Explain in Detail**
APC	advanced pancreatic cancer
GEM	gemcitabine
FX	FOLFIRINOX
GnP	gemcitabine plus nab-paclitaxel
mFX	modified FOLFIRINOX
5-FU	5-fluorouracil
OS	overall survival
DCR	disease control rate
PFS	progression free survival
RR	response rate
5-FU	fluorouracil
FN	febrile neutropenia
WBC	white blood cell count
CI	confidence interval
S-1	the combination drug of tegafur, gimeracil and oteracil
ICI	immune check point inhibitor
PARP	poly ADP-ribose polymerase
